# Role of Dietary Ceramide 2-Aminoethylphosphonate on Aberrant Crypt Foci Formation and Colon Inflammation in 1,2-Dimethylhydrazine-Treated Mice: A Comparison with the Role of Sphingomyelin

**DOI:** 10.3390/metabo15030147

**Published:** 2025-02-21

**Authors:** Shinji Yamashita, Wakaba Yutani, Maho Sugimoto, Kazuo Miyashita, Mikio Kinoshita

**Affiliations:** 1 Department of Life and Food Sciences, Obihiro University of Agriculture and Veterinary Medicine, Obihiro 080-8555, Japan; syamashita@obihiro.ac.jp (S.Y.);; 2 Center for Industry-University Collaboration, Obihiro University of Agriculture and Veterinary Medicine, Obihiro 080-8555, Japan; kmiyashita@do-bunkyodai.ac.jp; 3 Department of Health and Nutrition Sciences, Hokkaido Bunkyo University, Eniwa 061-1449, Japan

**Keywords:** cancer, ceramide, 2-aminoethylphosphonate, inflammation, intestine, phosphocholine, sphingolipid, sphingomyelin

## Abstract

**Background**: Ceramide 2-aminoethylphosphonate (CAEP), a major sphingolipid class in mollusks, possesses unique structures that are not observed in other sphingolipids. CAEP has a carbon–phosphorus bond and unusual long-chain bases (LCBs). CAEP has been reported to exhibit nutritional functions, such as improving skin conditions and promoting cholesterol metabolism. Objectives: In this study, we investigated the role of dietary CAEP in the formation of aberrant crypt foci (ACF) and colon inflammation induced by 1,2-dimethylhydrazine (DMH) in mice. **Methods**: Five-week-old female Bagg Albino/c mice were divided into four groups (*n* = 11), which were treated with the respective experimental diet and DMH intraperitoneal injection nine times for ten weeks. The results obtained after administering CAEP were compared with those obtained after administering sphingomyelin (SPM), which is a major sphingolipid in mammal-derived foods. **Results**: The predominant LCB in the octopus-extracted CAEP was determined as hexadeca-4-sphingenine. Dietary CAEP suppressed the formation of ACF, and egg yolk-derived dietary SPM exerted a higher suppressive effect on the formation of ACF. Additionally, dietary CAEP suppressed the DMH-decreased expressions of two inflammation-related cytokines in the colon mucosa, whereas dietary SPM normalized the expressions of two cytokines different from those suppressed by CAEP. **Conclusions**: CAEP provides intestinal protection, with effects that differ from those of SPM. The polar head groups or LCBs in sphingolipids are important for determining their nutritional function in the intestine. The study findings contribute toward the understanding of the nutritional benefits of sphingolipids in daily diets or supplements in maintaining intestinal health.

## 1. Introduction

The incidence of intestinal impairments, such as colon cancer and inflammatory bowel disease (IBD), has recently increased in East Asian countries, while cases in Western countries continue to remain high [1,2]. Patients with IBD often undergo long-term treatments that increase the risk of developing colon cancer [3]. Previous reports suggest that environmental factors, along with dietary nutrients, contribute to IBD pathogenesis. Diets rich in fats and proteins, which are common in the Western world and in countries with similar lifestyles, have been identified as risk factors for the development of IBD. However, some dietary nutrients can lower the risk of IBD [4,5]. Epidemiological studies have indicated a strong association between colon cancer and diets [6].

Sphingolipids are primarily located within the cell and vacuole membranes of most eukaryotes and some prokaryotes. Sphingolipids are a class of lipids that play important structural and functional roles in cells and tissues [7]. Sphingolipids have a ceramide (Cer) structure composed of a long-chain base (LCB) and an amide-linked fatty acid. Sphingolipids, which are present in most foods, primarily exhibit complex structures with a polar head group attached to Cer, rather than free Cer. The complex sphingolipid class is dependent on the food source. Sphingomyelin (SPM), glucosylceramide (GlcCer), and ceramide 2-aminoethylphosphonate (CAEP) are major classes of sphingolipids found in mammals, plants and fungi, and mollusks, respectively. SPM, GlcCer, and CAEP have complex structures with phosphocholine, glucose, and 2-aminoethylphosphonate attached to Cer, respectively.

CAEP has a unique structure that is not observed in other sphingolipid classes. CAEP is a phosphonolipid, which means it contains a carbon–phosphorus bond [8]. CAEP contains unique and unusual LCBs, including hexadeca-4-sphingenine (d16:1) and 2-amino-9-methyl-4,8,10-octadecatriene-1,3-diol (d19:3) [9]. Since the 1960s, several studies have been conducted on examining the digestion and absorption of SPM and GlcCer as components in food [10,11]. Previous studies have shown that SPM and GlcCer have various nutritional benefits, including improvement of skin and colon impairment [7,12]. SPM and GlcCer also suppress lipid absorption and improve lipid metabolism in Zucker fatty rats fed a high-fat diet, which is used as an obesity model [13]. However, few studies have investigated the nutritional value of CAEP. Recent studies have reported that CAEP is suggestively digested to Cer by the enzyme, sphingomyelinase, in the same way as SPM [14] and subsequently, Cer is hydrolyzed to LCBs by ceramidase. The unique and unusual LCBs are absorbed into lymph in the same way as normal LCBs; however, the absorption of d19:3 is low [15]. Subsequently, the absorbed LCBs are resynthesized, mainly in complex sphingolipids. In hairless mice, dietary CAEP derived from the skin of jumbo flying squid has been reported to improve the skin barrier function to the same extent as that improved by maize-derived GlcCer [16]. Additionally, as compared to SPM derived from chicken egg yolk, dietary CAEP derived from internal organs of scallop has been reported to be more advantageous for improving cholesterol metabolism in mice [17].

In contrast, complex sphingolipids are hardly digested and absorbed compared to other lipids [10,11,15]; therefore, themselves and their digests, including Cer and LCBs, are metabolized by digestive enzymes and gut microbiota on the colon, and they can affect colon conditions. Administration of SPM and glycosphingolipids, including GlcCer, has been reported to suppress the formation of ACF in the colons of 1,2-dimethylhydrazine (DMH)-treated rodents, serving as a precursor model for preventing colon cancer [18,19,20]. Dietary GlcCer also alleviates colon inflammation induced by DMH [21]. However, to the best of our knowledge, the effect of dietary CAEP on ACF formation and colon inflammation has not yet been explored.

In this study, we investigated the effect of dietary CAEP in comparison with SPM, a sphingolipid-bearing phosphate in the same way as CAEP, on ACF formation and colon inflammation. Mice were treated with DMH for ACF formation and chronic colon inflammation, and the effects of diets supplemented with CAEP on colon impairment were evaluated based on the number of ACF and expression levels of inflammation-related cytokines.

## 2. Materials and Methods

### 2.1. Preparation of CAEP from Octopus

CAEP was prepared from the boiled legs of the North Pacific giant octopus (*Enteroctopus dofleini*), which was purchased from a market in Hokkaido, Japan. At first, a 14 kg octopus was homogenized and freeze-dried to obtain 2 kg of dried powder. The crude lipid fraction was extracted from the prepared powder using a solution of chloroform, methanol, and water. Subsequently, alkaline-stable lipid fractions, including CAEP sphingolipids, were obtained by treating the crude lipid fraction with 0.4 M potassium hydroxide in the methanol solution at 38 °C for 2 h [22]. The extracted CAEP was purified using silica column chromatography with a stepwise gradient of chloroform/methanol solution [23]. The purity of CAEP was confirmed by thin-layer chromatography (TLC) using ninhydrin reagent and 50% sulfate. Finally, the prepared fraction weighed 3.68 g, and the CAEP purity was >90% (Figure S1). The used reagents were of extra-pure grade (Fujifilm Wako Pure Chemical Co., Osaka, Japan).

### 2.2. Analysis of CAEP Purified from Octopus

The fatty chain composition of the prepared CAEP was analyzed using a previously reported method [24,25]. At first, CAEP was treated with 1 M hydrogen chloride in the methanol/water solution at 70 °C for 18 h to obtain LCBs and fatty acid methyl esters. The LCBs were converted into fatty aldehydes using sodium periodate. The prepared fatty aldehydes and fatty acid methyl esters were analyzed using gas chromatography–mass spectrometry (GC–MS) (GC-2030 and GCMS-QP2020NX instruments, Shimadzu, Kyoto, Japan) with a CP-Sil 88 GC column (50 m × i.d 0.20 mm, df 0.25 μm, Agilent, CA, USA). Egg yolk-derived SPM (≥98% on TLC, Nagara Science Co., Ltd., Gifu, Japan) was also analyzed using GC–MS. Additionally, the CAEP species were converted into trimethylsilyl (TMS) derivatives using 1-TMS imidazole (Fujifilm Wako Pure Chemical Co., Osaka, Japan) and analyzed using a GC–MS instrument equipped with a ULBON HR-1 (5 m × i.d. 0.20 mm, df 0.25 μm, Shinwa Chemical Industries, Kyoto, Japan) [26].

### 2.3. Animals

Four-week-old female Bagg Albino/c mice were obtained from Japan SLC, Inc. (Shizuoka, Japan) and housed in isolator cages at 22 °C under a 12 h light/dark cycle. After one week of acclimatization, the mice were randomly divided into four groups (*n* = 11 per group; 3–4 mice per cage): control group, DMH, DMH + CAEP, and DMH + SPM groups. The control group was administered a control diet with intraperitoneal (i.p.) vehicle administration. The control diet was based on the AIN-76 rodent diet, which does not contain sphingolipids, and butylated hydroxytoluene (0.1 g/kg diet) as an antioxidant was added to it [27]. The DMH group was administered the control diet with i.p. DMH administration. The DMH + CAEP group was administered the CAEP diet with i.p. DMH administration. The CAEP diet contained 1 g/kg CAEP. The DMH + SPM group was administered the SPM diet with i.p. DMH administration. The SPM diet contained 1 g/kg SPM. In previous studies, diets containing sphingolipids (0.05–5.0 g/kg) suppress the ACF formation in the colons of DMH-treated rodents, and the most frequent amount used is 1.0 g/kg [18,19,20,28,29]. The feeding period of the experimental diet was ten weeks, and each mouse was i.p. intraperitoneally administered 15 mg/kg body weight of DMH hydrochloride (Tokyo Chemical Industry Co., Ltd., Tokyo, Japan) once a week for a total of nine times. DMH hydrochloride was administered for the first and last time on days 7 and 63, respectively. The mice were sacrificed one week after the last injection. The colons of five mice from each group were used as specimens, and the remaining colons were prepared for protein assays.

### 2.4. ACF Identification

**Figure 1:**
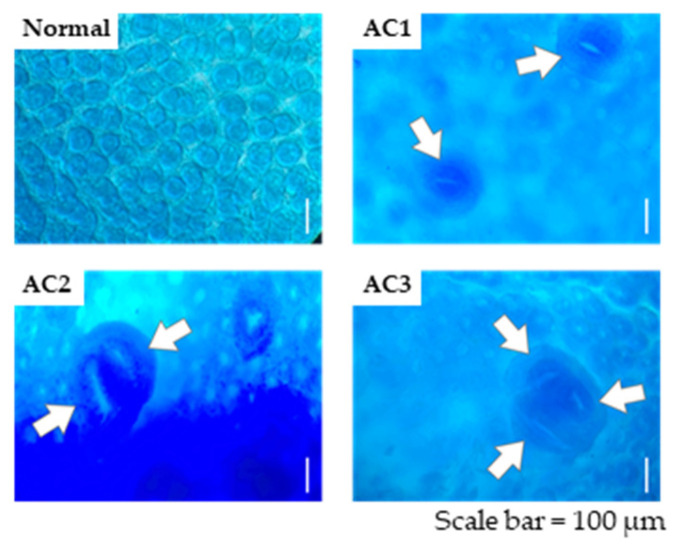
Topographic view of methylene blue-stained crypts in the colon showing different numbers of ACs, with AC1, AC2, and AC3 denoting 1 crypt, 2 crypts, and ≥3 crypts, respectively. The arrows in the pictures indicate ACs contained in ACF. ACF, aberrant crypt foci; AC, aberrant crypt.

ACF in the large intestinal crypts were identified and quantified as described in previous reports [30,31]. The colons were prepared as specimens to determine the quantity of ACF. The large intestine was excised from mice under a mixture of three anesthetic agents, domitor, dormicum, and vetorphate, and a portion of the intestine from the cecum to the vent was separated and rinsed with cold saline. The colon was fixed overnight in phosphate-buffered saline containing 4% paraformaldehyde and subsequently stained with 0.3% methylene blue solution in saline for 30 min at 20 °C. ACF were counted throughout the entire colon using a light microscope (BX-51; Olympus, Tokyo, Japan). ACF in the colon were classified according to the number of aberrant crypts (ACs), with AC1, AC2, and AC3 denoting 1 crypt, 2 crypts, and ≥3 crypts, respectively (Figure 1).

### 2.5. Inflammation Assay

The levels of inflammation-related cytokines in the mice colon mucosa were determined using the Mouse Cytokine Array kit (R&D Systems, Minneapolis, MN, USA). Owing to the limitations of the kit, four samples from each group were analyzed. The protein expression of the colon mucosa homogenates was determined according to the instructions provided by the manufacturer of the array kits. The following cytokines were detected: B lymphocyte chemoattractant (BLC), chemokine (C-C motif) ligand 1 (I-309), complement component 5a (C5/C5a), eotaxin, granulocyte macrophage colony-stimulating factor (GM-CSF), interferon-γ (IFN-γ), interferon-γ-induced protein 10 (IP-10), interleukin-1ra (IL-1ra), IL-2, IL-3, IL-4, IL-5, IL-6, IL-7, IL-10, IL-12, IL-13, IL-16, IL-17, IL-23, IL-27, metallopeptidase inhibitor 1 (TIMP-1), monocyte chemoattractant protein 2 (MCP-2), macrophage inflammatory protein-1β (MIP-1β), macrophage inflammatory protein-1α (MIP-1α), monokine induced by gamma interferon (MIG), monocyte chemotactic protein 5 (MCP-5), monocyte-specific cytokine MCP-1 (JE), macrophage colony-stimulating factor (M-CSF), neutrophil-activating protein 3 (KC), interferon-inducible T cell alpha chemoattractant (I-TAC), regulated on activation, normal T cell expressed and secreted (RANTES), soluble intercellular cell adhesion molecule-1 (sICAM-1), stromal cell-derived factor 1 (SDF-1), thymus and activation regulated chemokine (TARC), triggering receptor expressed on myeloid cells 1 (TREM-1), and TNF-α. The protein content of the specimens was measured using the DC Protein Assay kit (Bio-Rad, Hercules, CA, USA).

### 2.6. Statistical Analysis

Data among groups were analyzed by one-way analysis of variance using Tukey’s test. Differences were considered statistically significant at *p* < 0.05. All data were analyzed using BellCurve for Excel (Social Survey Research Information Co., Ltd., Tokyo, Japan) and were expressed as the mean ± standard error of the mean.

## 3. Results

### 3.1. Fatty Chain Composition of Sphingolipids—CAEP and LCB

**Figure 2:**
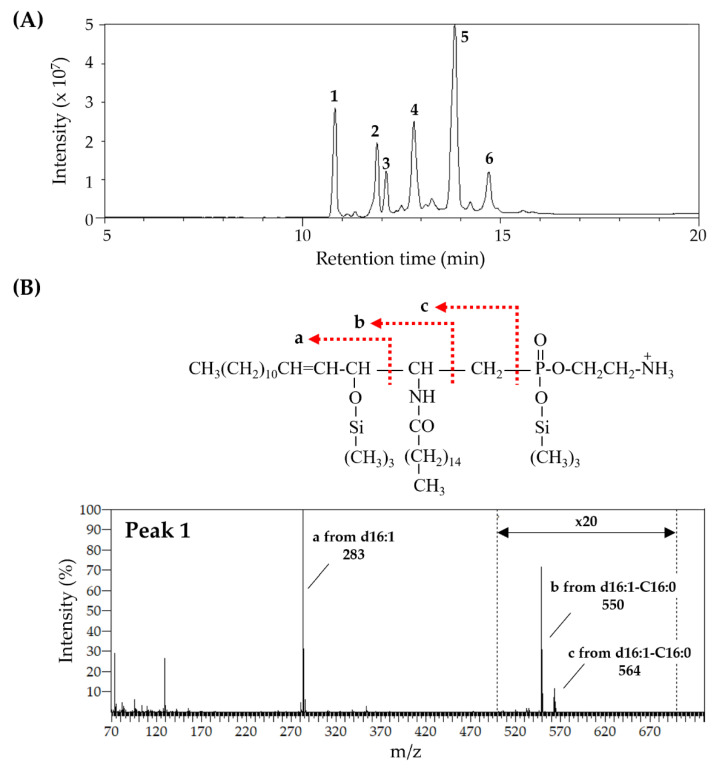
GC–MS analysis of the TMS derivative of CAEP prepared from octopus. (**A**) Total ion chromatogram of CAEP treated with TMS. The respective peaks correspond to the following species: (1) d16:1-C16:0, (2) d16:1-C18:0 and d18:1-C16:0, (3) d19:2-C16:0, (4) d16:1-C20:1, (5) d16:1-C22:1 and d18:1-C20:1, and (6) d16:1-C24:1 and d18:1-C22:1. (**B**) Structure and mass spectrum of peak 1 in (**A**). a, b, and c in the mass spectrum correspond to the respective fragment ions from the structure. CAEP, ceramide 2-aminoethylphosphonate; GC–MS, gas chromatography–mass spectrometry; TMS, trimethylsilyl.

The prepared CAEP is predominantly composed of d16:1 as an LCB and palmitate (C16:0) and 13-docosenoate (C22:1) as fatty acids (Table 1). In addition, a trace level of d19:3 is observed in the CAEP. In contrast, SPM is predominantly composed of sphingosine (d18:1), a usual LCB in mammals, and C16:0. Analysis of CAEP TMS-derivatives indicates the presence of d16:1-C16:0, d16:1-C18:0, d18:1-C16:0, d19:2-C16:0, d16:1-C20:1, d16:1-C22:1, d18:1-C20:1, d16:1-C24:1, and d18:1-C22:1 species (Figure 2 and Figure S2).

### 3.2. ACF Formation

**Figure 3:**
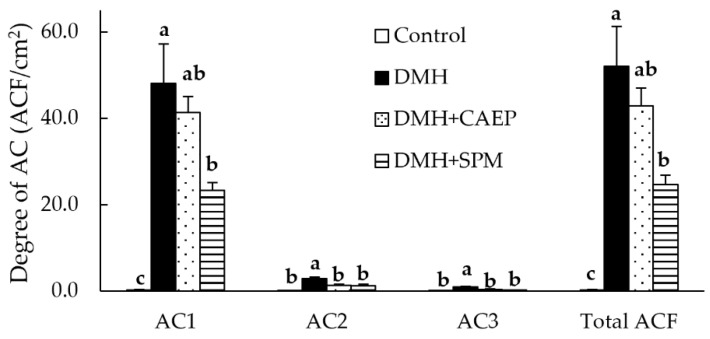
Effect of dietary CAEP and SPM on ACF formation in mice colons after nine injections of DMH. Data are represented as the mean ± standard error of the mean (*n* = 5). Different letters (a, b, and c) indicate significant differences among the experimental groups at *p* < 0.05, estimated using one-way analysis of variance with Tukey’s test. ACF, aberrant crypt foci; AC, aberrant crypt; CAEP, ceramide 2-aminoethylphosphonate; DMH, 1,2-dimethylhydrazine; SPM, sphingomyelin.

As shown in Figure 3, the number of ACs and total ACF are significantly higher in the DMH group than in the control group. In contrast, the DMH + CAEP group shows significantly lower numbers of AC2 and AC3 than those in the DMH group. The DMH + SPM group shows significantly lower numbers of AC1, AC2, AC3, and total ACF than those in the DMH group. ACF formation in the DMH + SPM group is not significantly different from that in the DMH + CAEP group. As compared to the control group, the DMH-treated groups exhibit significantly lower body weights after nine weeks of DMH treatment (Table 2). The DMH group exhibits a lower liver weight than that exhibited by the control group. No differences in feed intake, colon length, or spleen and fecal weights are observed among the different groups.

### 3.3. Expressions of Inflammation-Related Cytokines in the Colon

**Figure 4:**
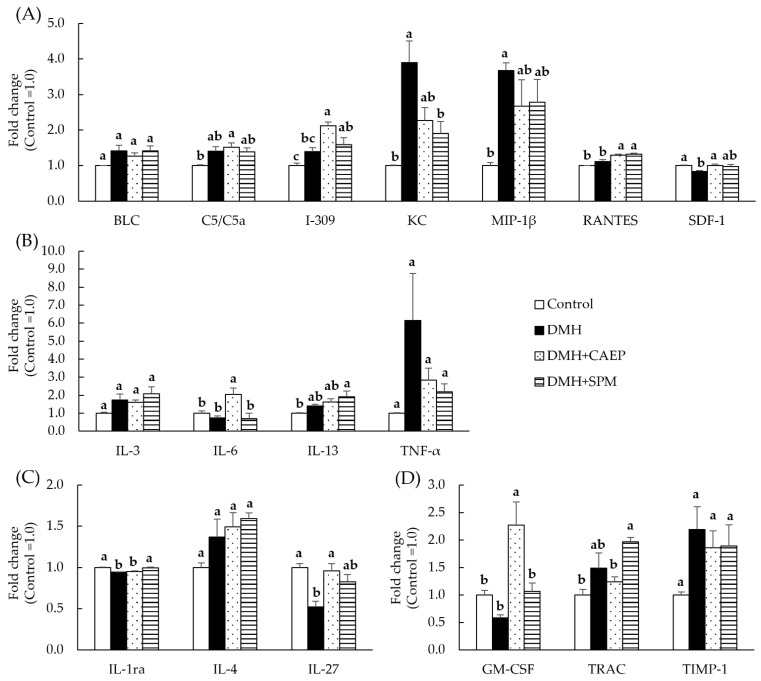
Effect of dietary CAEP and SPM on the expression of inflammation-related cytokines in the colon mucosa of mice after nine injections of DMH. Expression of (**A**) chemokines, (**B**) inflammatory cytokines, (**C**) anti-inflammatory cytokines, and (**D**) other cytokines in various experimental groups. Data are represented as the mean ± standard error of the mean (*n* = 4). Different letters (a and b) indicate significant differences among the experimental groups at *p* < 0.05, determined using one-way analysis of variance with Tukey’s test. Please refer to the Material and Methods Section 2.5 for abbreviations of inflammation-related cytokines. CAEP, ceramide 2-aminoethylphosphonate; DMH, 1,2-dimethylhydrazine; SPM, sphingomyelin.

Figure 4 and Figure S3 indicate the expressions of inflammation-related cytokines in the colon mucosa of mice. Figure 4A–D shows that the DMH treatment markedly increases the expression of two chemokines (KC and MIP-1β) and significantly decreases the expression of one chemokine (SDF-1) and two anti-inflammatory cytokines (IL-1ra and IL-27). Additionally, DMH treatment increases the expressions of BLC (*p* = 0.097), TNF-α (*p* = 0.085), and TIMP (*p* = 0.094).

Dietary CAEP significantly suppresses the DMH-induced decrease in the expressions of SDF-1 and IL-27 and attenuates the DMH-induced increase in the expression of KC (*p* = 0.051). Additionally, the expressions of I-309, RANTES, IL-6, and GM-CSF in the DMH + CAEP group are markedly higher than those in the control and DMH groups. The expression of C5/C5a in the DMH + CAEP group is significantly higher than that in the control group. In contrast, dietary SPM normalizes the DMH-induced expression of KC and IL-1ra. Dietary SPM also suppresses the DMH-induced decrease in the expression of IL-27 (*p* = 0.059). Moreover, the expression of RANTES in the DMH + SPM group is significantly higher than that in the control and DMH groups, and the expressions of I309, IL-13, and TRAC in the DMH + SPM group are markedly higher than those in the control group. The expressions of BLC (*p* = 0.095), IL-3 (*p* = 0.051), and IL-4 (*p* = 0.057) in the DMH + SPM group are higher than those in the control group.

## 4. Discussion

Dietary SPM and glycosphingolipids are complex sphingolipids that suppress ACF formation [18,19,20]. Previous studies have suggested that complex sphingolipids are metabolized to Cers and LCBs in the intestine, suppressing aberrant increases in abnormal intestinal cells [19,32,33,34]. An in vitro study on intestinal epithelial cells reported that sphingolipids metabolize into phosphorylated LCBs in the presence of an inflammatory stress, which alleviates inflammatory injury [35]. In this study, we found that CAEP, a major sphingolipid class in mollusks, suppresses ACF formation; however, the extent of suppression by CAEP is lower than that exhibited by egg yolk-derived SPM. Additionally, CAEP and SPM are observed to normalize the DMH-affected expressions of other cytokines, as shown in Figure 4.

The observed differences in the suppressive effects of CAEP and SPM on ACF formation can be attributed to two factors.

First, SPM and its metabolites may reach the colon in a higher amount than that of CAEP. Compared to SPM, which has an LCB composition of 97.9% d18:1 (possibly egg yolk-derivative), squid skin-derived CAEP, which has an LCB composition of 41.4% d19:3 and 28.6% d16:1, hydrolyzes at a faster rate at pH 7.2 in the small intestinal mucosa of mice [14]. Additionally, the LCB absorption ratio is higher for d18:1 and d16:1 than that for d19:3 [15]. Thus, CAEP—especially the one that mainly constitutes d16:1—can be anticipated to show a lower suppressive effect on ACF formation than that shown by SPM. Therefore, CEAP administration can be anticipated to result in a higher and a lower level of LCBs in the small and large intestine, respectively, than those observed after SPM administration.

Second, CAEP and SPM themselves are likely to exhibit significantly different effects on the colon. Previously, a comparison of the effects of administering Cer and GlcCer, both of which were composed of the same LCB and fatty acid species, in DMH-treated mice showed that the DMH + GlcCer group suppresses ACF formation by the same extent as that by the DMH + Cer group, regardless of the different Cer levels observed in their feces [29]. Notably, only the DMH + GlcCer group suppresses colon inflammation [29], which is associated with the formation of colon cancer and ACF in humans and the DMH model [21,36].

In conclusion, complex sphingolipids have different nutritional functions depending on their structures; therefore, along with the metabolites, Cers and LCBs, CAEP and SPM may also impact ACF formation. Dietary CAEP and SPM normalize the expressions of inflammation-related cytokines induced by DMH in the colon mucosa; however, the normalized cytokines differ depending on the sphingolipid class. Additionally, CAEP and SPM increase the expressions of inflammatory cytokines IL-6 and IL-13, respectively. Since dietary CAEP and SPM suppress the DMH-induced expressions of apoptosis-related proteins, such as cleaved-caspase-3—an effector caspase, in the colon mucosa (Figure S4)—they alleviate colon inflammation by different mechanisms. Previous studies have reported that the suppressive effects of dietary sphingolipids on ACF formation are essentially the same regardless of class and LCB composition [19,20]. However, a possibility still exists that the suppressive mechanisms of dietary sphingolipids on ACF are structure-dependent. A possibility exists that the LCB composition of sphingolipids may affect the nutritional functions. Administration of egg yolk-derived SPM accelerates inflammatory injury in the colon of mice treated with dextran sulphate sodium (DSS) and IL-10 negative mice used as a spontaneous colitis model [37]. In contrast, bovine milk-derived dietary SPM, bearing the strange LCB composition of 44.4% d18:1, 15.1% d16:1, and 10.7% d18:1 (branched) [38], suppresses colonic inflammatory lesions in mice treated with DSS in combination with azoxymethane [39]. Milk-derived SPM alleviates an increase in serum levels of lipopolysaccharide (LPS), caused by altering gut microbiota, in mice fed a high-fat diet, whereas the effect of egg yolk-derived SPM is not observed [40]. Moreover, LCBs ameliorate LPS-induced injury in intestinal epithelial cells in vitro, and the extent differs depending on the LCB structure [35]. A comparative study on in vivo intestinal metabolism of complex sphingolipids is required to further validate the study findings. The presence of abundant usual SPM species in mammals makes it harder to analyze the results. More research focusing on the utilization of isotope-labeling [10] or SPM-bearing non-mammal-type LCBs (e.g., ascidian derivatives [41]) is needed.

Highlighting the relationship between intestinal impairments and gut microbiota, for example, *Blautia coccoides* abundance decreases in the gut of subjects with colon cancer and IBD [42,43], and *Blautia coccoides* accelerates colonic mucosal formation via secretion of short chain fatty acids [44] and is associated with production of the anti-inflammatory cytokine IL-10 in immune cells [45]. GlcCer has been reported to increase some Gram-positive bacteria, including *Blautia coccoides*, in both in vivo and in vitro by enhancing tolerance to bile acids [46,47]. Additionally, dietary excess fat as well as bile acids alter gut microbiota and aggravate intestinal inflammation [48,49]. CAEP and SPM exert bile acid binding capacity in vitro and affect the gut microbiota in mice fed a high-fat diet [17,40,50]. Therefore, it is also expected that dietary sphingolipids improve gut microbiota to alleviate intestinal impairments. Future studies are needed to more clearly identify the role of dietary sphingolipids for the prevention of intestinal impairments.

## 5. Conclusions

In this study, we investigated the role of dietary CAEP in the formation of ACF and colon inflammation induced by DMH in mice. The administration of CAEP, a sphingolipid with unique and unusual structures, suppresses ACF formation and colon inflammation. The polar head groups and/or LCBs of sphingolipids are important for determining their impact on the intestine. The study findings advance the understanding of the role of sphingolipids in maintaining intestinal health.

## Figures and Tables

**Table 1:** Fatty chain composition of CAEP and SPM.

Fatty Acid	CAEP	SPM	LCB	CAEP	SPM
	mol%			mol%	
C16:0	34.2	80.2	d16:1	84.2	nd
C18:0	10.2	7.9	d18:0	nd	6.2
C20:1	12.0	nd	d18:1	14.1	93.8
C22:1	37.6	nd	d19:2	1.7	nd
C24:1	2.8	4.6	d19:3	trace	nd
Others	3.3	7.3			

CAEP, ceramide 2-aminoethylphosphonate; nd, not detected; SPM, sphingomyelin.

**Table 2:** Weight of the body, liver, and spleen, along with colon lengths, of the mice in various groups after nine weeks of DMH treatment.

	Control	DMH	DMH + CAEP	DMH + SPM
Final body weight (g)	22.33 ± 0.34 a	20.37 ± 0.38 b	20.73 ± 0.35 b	20.76 ± 0.15 b
Body weight gain (g/9 weeks)	3.92 ± 0.31 a	2.05 ± 0.28 b	1.56 ± 0.20 b	1.83 ± 0.26 b
Feed intake (g/9 weeks) *	166.04 ± 0.06 a	165.05 ± 5.11 a	174.97 ± 3.52 a	178.09 ± 0.37 a
Liver weight (g)	0.93 ± 0.03 a	0.81 ± 0.03 b	0.84 ± 0.03 ab	0.86 ± 0.02 ab
Spleen weight (g)	0.12 ± 0.01 a	0.12 ± 0.00 a	0.13 ± 0.01 a	0.13 ± 0.01 a
Colon length (cm)	10.75 ± 0.38 a	11.36 ± 0.53 a	11.31 ± 0.40 a	10.80 ± 0.55 a
Dry feces (g/2 days) *	0.28 ± 0.01 a	0.33 ± 0.03 a	0.36 ± 0.06 a	0.37 ± 0.01 a

Data are represented as the mean ± standard error of the mean (*n* = 11 or 3). Asterisk-marked items are calculated based on three data points and are determined as the amount per mouse because each cage housed only three or four mice. Feces were collected two weeks before the dissection. Different letters (a and b) indicate significant differences among experimental groups at *p* < 0.05, determined using one-way analysis of variance with Tukey’s test. CAEP, ceramide 2-aminoethylphosphonate; DMH, 1,2-dimethylhydrazine; SPM, sphingomyelin.

## Data Availability

The raw data supporting the conclusions of this article will be made available by the authors on request.

## Supplementary Materials

The following supporting information can be downloaded at https://www.mdpi.com/article/10.3390/metabo15030147/s1. Figure S1: CAEP on TLC analysis; Figure S2: Mass spectrum of the TMS derivatives of CAEP derived from octopus; Figure S3: Effect of dietary CAEP and SPM on the expression of inflammation-related cytokines for except cytokines shown in Figure 2 in the colon mucosa of mice after nine injections of DMH; Figure S4: Effect of dietary CAEP and SPM on the expressions of apoptosis-related proteins in mice colon mucosa after nine injections of DMH.
