# Supplementary material for: Role of Dietary Ceramide 2-Aminoethylphosphonate on Aberrant Crypt Foci Formation and Colon Inflammation in 1,2-Dimethylhydrazine-Treated Mice: A Comparison with the Role of Sphingomyelin
Source: Metabolites. 2025 Feb 21;15(3):147. doi: 10.3390/metabo15030147 (PMC11943771; doi:10.3390/metabo15030147)
Supplement: Supplementary file 1 [file metabolites-15-00147-s001.zip › metabolites-3461811-supplementary.pdf]

Supplementary Materials

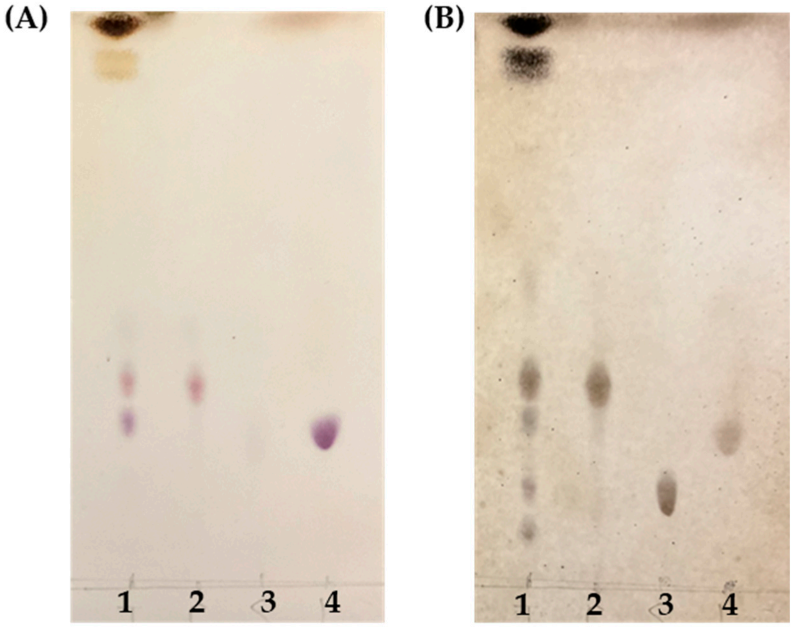

Figure S1. CAEP on TLC analysis. TLC results using the mobile phase chloroform/methanol/water (65:25:4) in (A) ninhydrin and (B) 50% sulfate as detection reagents. (1) Octopus-extracted lipids after alkali-treatment, (2) octopus-derived CAEP, (3) egg yolk-derived SPM, and (4) lyso-phosphatidylethanolamine. CAEP, ceramide 2-aminoethylphosphonate; SPM, sphingomyelin; TLC, thin-layer chromatography.

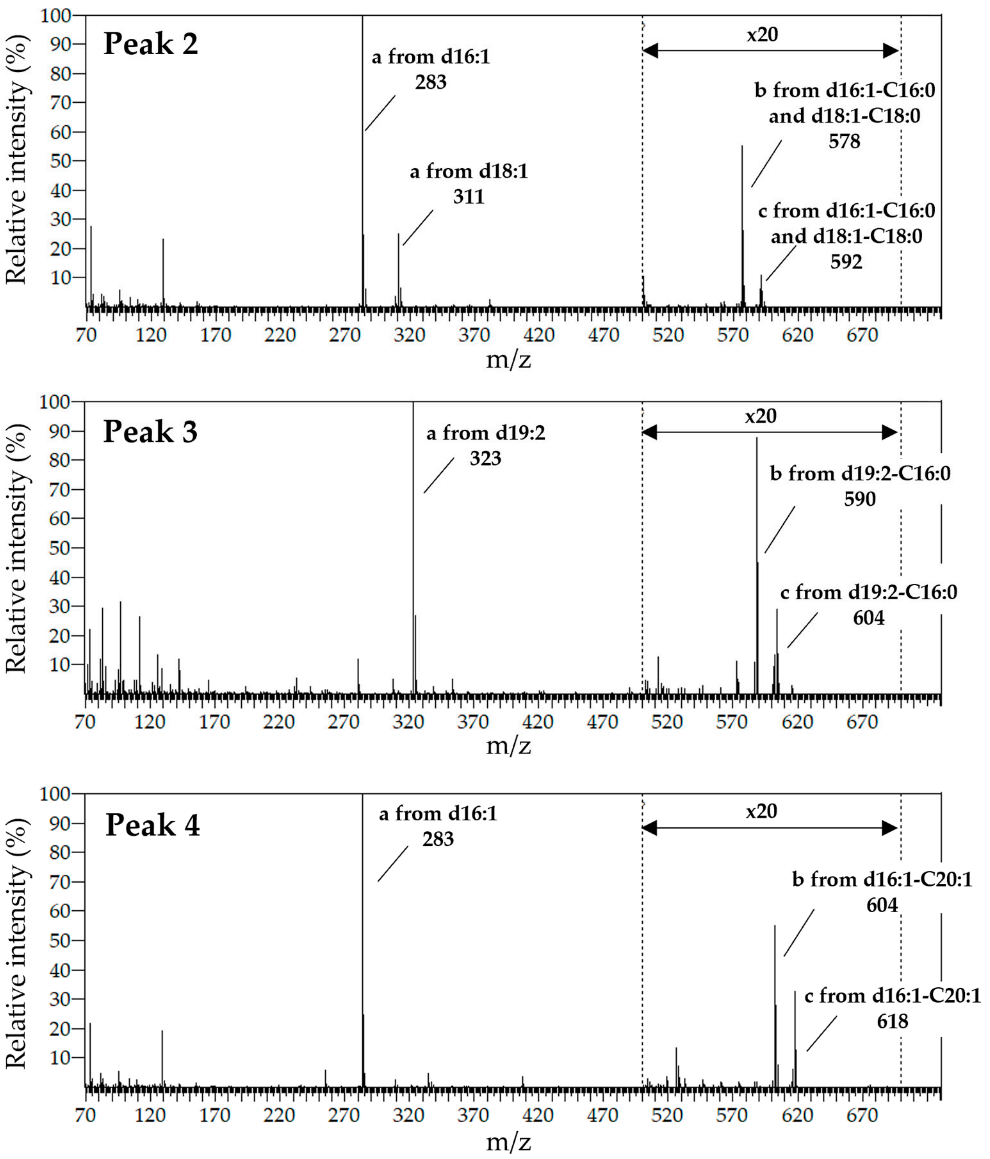
Peak 2
a from d16:1
283
a from d18:1
311
x20
b from d16:1-C16:0
and d18:1-C18:0
578
c from d16:1-C16:0
and d18:1-C18:0
592
Peak 3
a from d19:2
323
x20
b from d19:2-C16:0
590
c from d19:2-C16:0
604
Peak 4
a from d16:1
283
x20
b from d16:1-C20:1
604
c from d16:1-C20:1
618
Relative intensity (%)
m/z

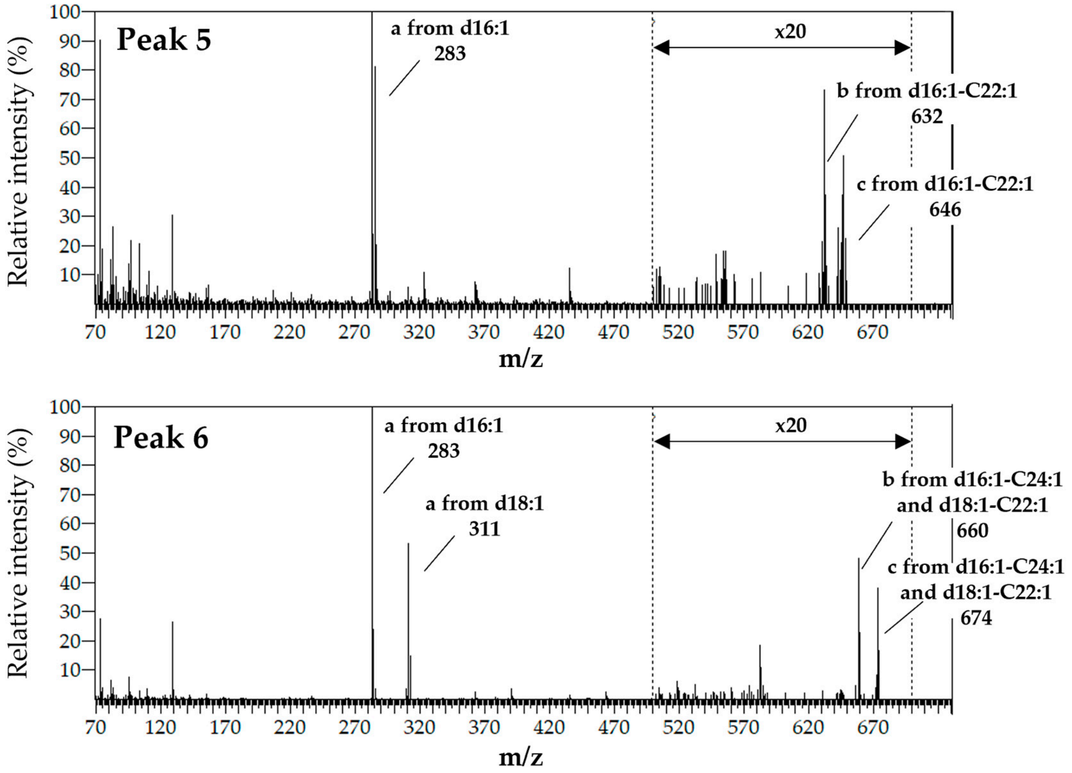

Figure S2. Mass spectrum of the TMS derivatives of CAEP derived from octopus. These mass spectrums correspond to the relative peaks 2-6 in Figure 1A. Please refer to Figure 1B for a, b, and c fragment ions from the structure. CAEP, ceramide 2-aminoethylphosphonate; TMS, trimethylsilyl.

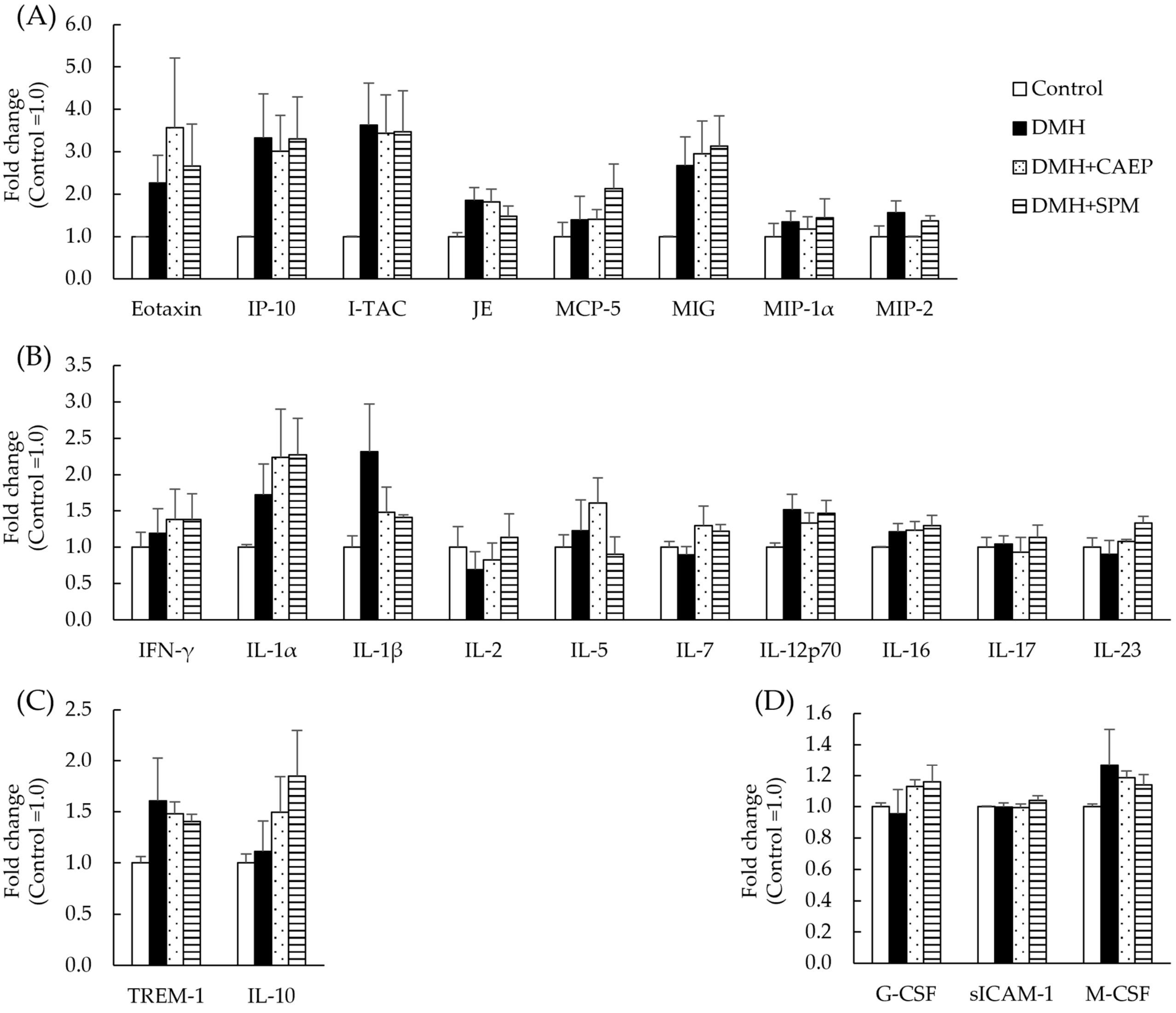

Figure S3. Effect of dietary CAEP and SPM on the expression of inflammation-related cytokines for except cytokines shown in Figure 2 in the colon mucosa of mice after nine injections of DMH. Expression of (A) chemokines, (B) inflammatory cytokines, (C) anti-inflammatory cytokines, and (D) other cytokines in various experimental groups. Data are represented as the mean ± standard error of the mean (n = 4). All cytokines have no significant differences among experimental groups at $p < 0.05$, determined using one-way analysis of variance with Tukey's test. Please refer to the *Material and Methods Section 2.5* for abbreviations of inflammation-related cytokines. CAEP, ceramide 2-aminoethylphosphonate; DMH, 1,2-dimethylhydrazine; SPM, sphingomyelin.

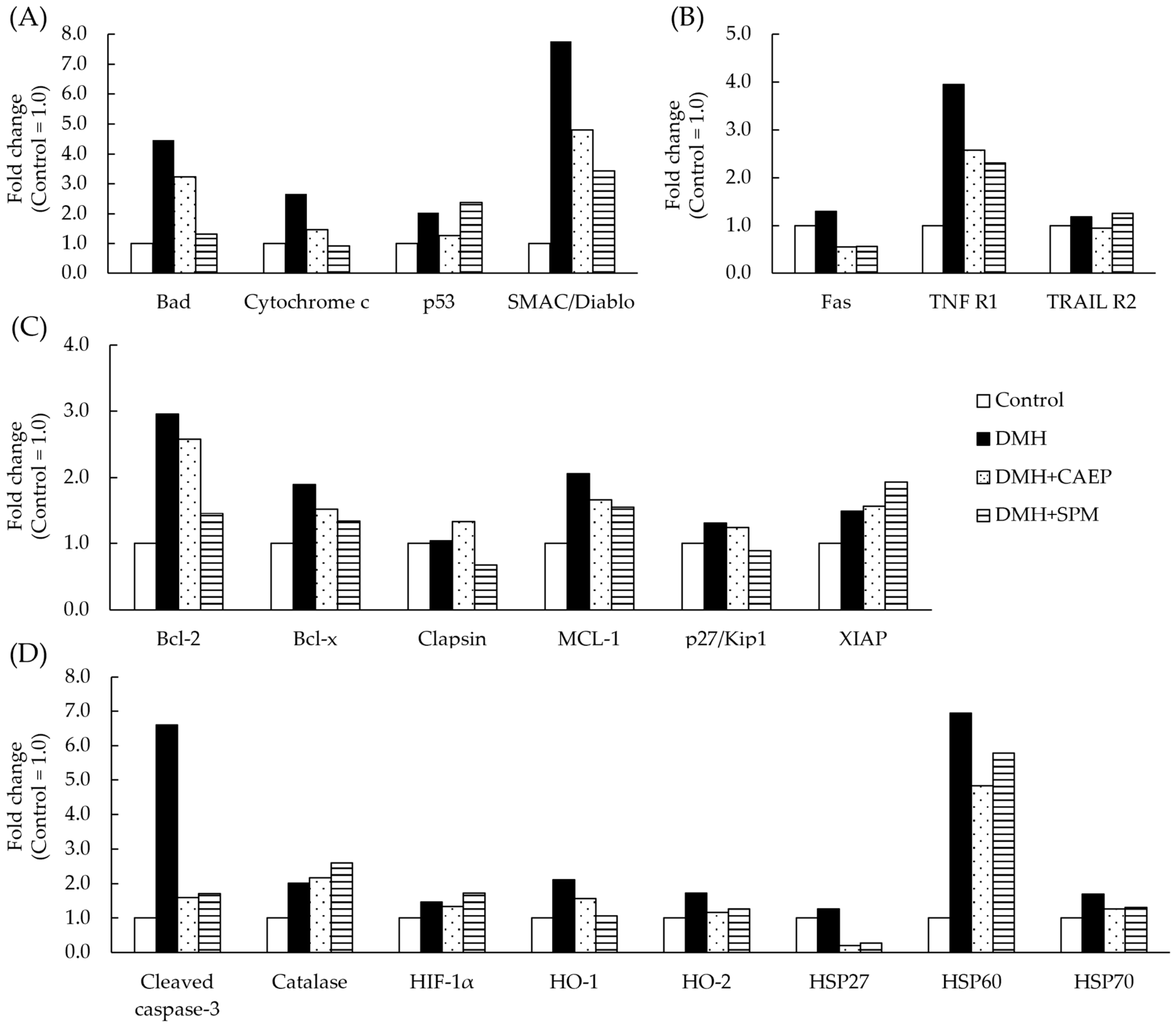

Figure S4. Effect of dietary CAEP and SPM on the expressions of apoptosis-related proteins in mice colon mucosa after after nine injections of DMH. Data are represented as the means (n = 2). Owing to the limitations of the samples, two samples from each group were analyzed. Expressions of (A) proapoptotic proteins, (B) death receptors, (C) antiapoptotic proteins, and (D) other apoptosis-related proteins. The levels of apoptosis-related proteins in the colon mucosa were determined using a Mouse Apoptosis Array Kit (R&D Systems, Minneapolis, MN, USA). Due to the limited availability of samples, only two samples were analyzed from each group. Protein contents of colon mucosa homogenates were detected according to the instructions provided by the manufacturer of the array kits. The following apoptosis-related proteins were detected: B-cell lymphoma 2 (Bcl-2), Bcl/leukemia x (Bcl-x), catalase, claspin, myeloid cell leukemia-1 (MCL-1), p27 cyclin-dependent kinase 4 inhibitor 1B (p27/Kip1), X-linked inhibitor of apoptosis (XIAP), Bcl-xL/Bcl-2 associated death promoter (Bad), cytochrome c, second mitochondria-derived activator of caspase/direct inhibitor of apoptosis-binding protein with low pI (SMAC/Diablo), fibroblast-associated (Fas), TNF receptor 1 (TNF R1), TNF-related apoptosis-inducing ligand receptor 2 (TRAIL R2), cleaved-caspase-3, p53, hypoxia-inducible transcription factor (HIF)-1α, heme oxygenase (HO)-1, HO-2, heat shock protein (HSP)27, HSP60, and HSP70. CAEP, ceramide 2-aminoethylphosphonate; DMH, 1,2-dimethylhydrazine; SPM, sphingomyelin.
